# Investigating Physical, Social, Emotional, and Health Frailties of Cancer Survivors after Cancer Treatment: The Urgent Call for Tailored Multidisciplinary Survivorship Plans in Italy

**DOI:** 10.3390/cancers16173080

**Published:** 2024-09-04

**Authors:** Stefania Moramarco, Luigi De Angelis, Laura Bernardini, Lorenza Marconi, Gaia Piunno, Simonetta Siciliano, Andrea Malizia, Ersilia Buonomo, Alessia Pesaresi, Angela Andreoli, Barbara Capotondi, Mario Roselli, Leonardo Palombi, Francesco Torino

**Affiliations:** 1 Department of Biomedicine and Prevention, University of Rome “Tor Vergata”, 00133 Rome, Italy; malizia@ing.uniroma2.it (A.M.); alessia.pesaresi@uniroma2.it (A.P.); angela.andreoli@uniroma2.it (A.A.); palombi@uniroma2.it (L.P.); 2 Department of Translational Research and New Technologies in Medicine and Surgery, University of Pisa, 56127 Pisa, Italy; 3 School of Specialization in Hygiene and Preventive Medicine, University of Rome “Tor Vergata”, 00133 Rome, Italy; 4 Medical Oncology Unit, Department of Systems Medicine, University of Rome “Tor Vergata”, 00133 Rome, Italytorino@med.uniroma2.it (F.T.)

**Keywords:** cancer, survivorship, frailty, overage, Italy

## Abstract

**Simple Summary:**

This pilot study aims to identify the physical, mental, social, psychological, and health needs encountered by cancer survivors in order to propose and facilitate appropriate and tailored responses. To the best of our knowledge, this is one of the first multidimensional studies investigating this topic in Italy. Data show that the quality of life of cancer survivors is affected by cancer and its treatment, reporting more frailties than the general population, especially those over 65 years old. These findings could help develop multidisciplinary planning of survivorship care for the transition of patients from oncological management to primary healthcare.

**Abstract:**

Background: Understanding the specific needs of cancer survivors is essential for healthcare policy. In Italy, dedicated studies are lacking, so we aimed to investigate the physical, mental, social, and health difficulties encountered by these patients. Methods: We conducted a cross-sectional study on breast or colorectal cancer survivors (people 5+ years free from it and its treatments) using an ad hoc survey including validated questionnaires (Grauer–Palombi, SF-36, PREDIMED). Participants were recruited within the Oncology Unit of the “Policlinico Tor Vergata”, Italy. Results: A total of 62 patients (80.6% females; years range: 37–87) agreed to be interviewed. A profile of cancer survivors was drafted: an overaged person with multiple co-morbidities, not well-nourished, adhering to the Mediterranean diet, reporting critical conditions as for physical and functional status. The mean number of co-morbidities was 3.6 ± 2.4 SD, with a statistically significant difference between age groups (under and over 65). Compared to the general population, the sample showed more frailties, especially when >65. The risk of having multimorbidity (four or more co-morbidities) significantly increased in those over 65 (OR: 4.72; CI: 1.43–15.59). Conclusion: There is an urgent need for survivorship care planning for the patient-centered continuum of care. Assessing and monitoring their specific needs will help propose appropriate and tailored responses.

## 1. Introduction

Cancer is a public health priority worldwide, with a growing incidence intensified also by the shift in age demographics. Nowadays, advances in cancer screening, diagnosis, and treatment have increased the possibility of making cancer a curable disease [1]. Survival for many types of cancer has improved over the years and tends to increase with time since diagnosis [2]. Currently, the probability of survival after cancer is nearly 40% for men and 50% for women, both in Europe and Italy [3].

The epidemiological picture of cancer in Italy during 2023 reports a total of 395,000 new cases of cancer. The highest number was detected for breast cancer (55,900 new cases), which is the most frequent cancer in women, accounting for 30% of all types of cancer. In 2023, 834,200 women people were living with breast cancer in Italy. The five-year net survival among breast cancer patients was particularly high (88%). Colorectal cancer was the second type of tumor for incidence in the general population (50,500 new cases) and per gender (23,700 new cases for females; 26,800 for males). Additionally, in 2023, 513,500 people were living with colorectal cancer (280,300 males; 233,200 females). Five-year net survival among colorectal cancer was 65% for men and 66% for women [3].

Cancer survivorship indicates the condition of a patient affected by cancer, but defining cancer survivorship may be difficult since various definitions have been reported in the literature. As a matter of fact, the literature contains more than twenty definitions of “cancer survivorship” [4]. Indeed, the term “cancer survivor” is commonly used by different persons, clinical and academic institutions, and political organizations, lacking however a unanimous and detailed definition. One of the most widely accepted definitions of cancer survivorship is that it is an ongoing process that commences at the time of diagnosis and continues throughout the remainder of the individual’s life. This definition underscores the importance of addressing the medical, psycho-social, and spiritual needs of cancer patients, necessitating continuous care and support. Differences exist not only about when a person becomes a survivor (e.g., at the time of diagnosis or after completing treatment) or even if people who are actively dying may be considered survivors but also whether healthy caregivers, family members, and friends of the cancer patient might also be considered survivors. Notably, some people who have been diagnosed with cancer reject the term “survivor” or disagree with some aspects included in the term [4].

Ideally, successful cancer treatment should be followed by a return to normal life. However, this process might be hindered by long-term and late effects of cancer and its treatment, as well as psycho-social issues [5], that might expose these people to a manifested frailty and specific needs. Long-term symptoms are commonly reported by patients even long after cancer treatment. As an example, pain, fatigue, depression, and emotional concerns are known symptoms that often co-occur and require concurrent management [6,7]. In the last decades, most of the research on cancer survivorship has focused on patients from the diagnosis to the completion of their follow-up period. Less is known about the condition of people who are free of cancer after the completion of their post-treatment 5-year follow-up. Cancer survivors require follow-up to manage the effects of cancer and its treatment, screen for recurrence or the appearance of new cancer, and coordinate care [8]. Many of them report that the adaptations required during survivorship are more challenging than those required during treatment [9] since long-term and delayed effects on individual health are still not well documented [10]. Therefore, this vulnerable group should require additional special attention, with empirical data on their health status and risk profiles, including determinants and influencing factors.

Remarkably, the European Commission’s Joint Action on Cancer Control has recommended tailored survivorship care plans that include guidelines for monitoring and maintaining cancer survivors’ health. A structured follow-up program for cancer survivors can prevent late complications or detect early signs and permit early treatment [11]. In response to this, in most recent years, surveys on cancer survivors have been growing, and many groups have developed various types of care plans to help improve the quality of care for survivors. However, many gaps remain [12] since this has not been happening in most countries worldwide with formal consensus statements and harmonized national guidelines for long-term follow-up [13].

For example, in Italy, we do not have a clear picture of breast and colorectal cancer long-term survivors’ specific constraints because there is currently a lack of detailed studies that describe accurately their health and social care needs. As a consequence, the development of a comprehensive person-centered survivorship planning is still lacking.

Therefore, a dedicated study aimed at this segment of the Italian population, whose needs still require to be better understood, was necessary. The choice to focus our study on patients affected by breast and colorectal cancer was made based on an epidemiological reason. These malignancies are among the most prevalent in Italy, and affected patients have among the most prolonged survival and highest cure rates. Moreover, the survivorship aspects of these patients are more extensively treated in the literature, offering the chance to better compare our findings with other experiences. Specifically, the objective of the present pilot study was to analyze and identify the physical, mental, and psychological needs, as well as the social difficulties encountered by those individuals, in order to facilitate and propose appropriate and tailored client-based responses in the future.

## 2. Materials and Methods

### 2.1. Study Design and Sample

Between June 2023 and February 2024, we conducted an observational cross-sectional study under the aegis of the Italian Cancer League (Lega Italiana per la Lotta contro i Tumori, LILT), consisting of data collected through a structured survey offered to a convenience sample of men and women who survived cancer.

Inclusion criteria were to have been previously diagnosed with breast or colorectal cancer and to have completed active oncological treatment at least 5 years before (adjuvant hormone therapy in breast cancer individuals lasting more than five years was allowed). Participants who met these criteria were randomly selected from the registry of the Medical Oncology Unit of the “Policlinico Tor Vergata”. We performed a systematic sampling with the evaluation of all the patients incoming on certain fixed days of the week who were contacted by phone by the medical staff to schedule an appointment for the follow-up visit. During the phone call, clients were informed about the study, and potential participants were given an appointment for the specific interview. Participation in the study was voluntary. All the clients were asked to complete a survey administered by physicians of the Hygiene and Preventive Medicine of the “University of Rome Tor Vergata”. Before participating in the survey, patients had to provide their informed consent. Patients were informed of their freedom to withdraw from the study at any time, without consequences for the examinations or follow-up visits planned. In compliance with the current Italian legislation on personal data (Legislative Decree 196/2003), each patient was assigned an alphanumeric identification code before starting the questionnaire administration to ensure anonymity. There were no incentives or rewards offered for taking part in the study. The study was approved by the Ethics Committee of the University of Rome “Tor Vergata” (Rome) (n. 126.22, 2022).

### 2.2. Software Development

The authors created an online ad hoc software that contained, in distinct sections, the survey used for the evaluation (see the following paragraph for the questionnaires’ details). The health staff accessed and administrated the survey via a mobile device (tablet). All answers were automatically transferred into a Google form. Some fields (e.g., Body Mass Index) were automatically calculated through a Google form plug-in (formfacade), which also allowed the automatic calculation of the scores obtained from each questionnaire. Data were saved to a CSV file that was automatically updated on cloud storage, accessible only by the administrators, and protected through a cybersecurity system.

### 2.3. Questionnaires

The first section was reserved for targeted socio-demographic information, anthropometric information, and health conditions.

Following a comprehensive review of the relevant literature, we selected validated questionnaires to depict the physical, social, emotional, and psychological conditions of the persons interviewed.

The first questionnaire selected was a validated Italian version [14] of the Grauer Functional Rating Scale (GFRS) for geriatric assessment [15]: the Grauer–Palombi Scale. It examines various aspects of physical, mental, and social health, providing a comprehensive view of the patient’s condition. Specifically, the first part of the scale focuses on the patient’s physical and mental conditions, including their medical history of diseases. The questions in the first part are assigned negative scores: the more the disability increases, the more the score becomes negative. The subsequent questions assess the patient’s functional ability in daily activities and the availability of social and community support, as well as their domestic and economic situation. The questions in the second part are assigned positive scores: the more the disability increases, the lower the scores that would be recorded. The final score is given by the sum of the singular scores and allows us to categorize patients into three groups:

- Category 1—score less than 20: frail patient (unable to live at home alone and requires intensive home assistance or hospitalization);
- Category 2—score between 20 and 40: pre-frail patient (can live at home with the support of day hospital services and/or assistance);
- Category 3—score greater than 40: robust patient (can live independently and does not require significant external help.

The second questionnaire was the 36-item short-form (SF-36) [16], a multidimensional validated tool designed to quantify health status and measure health-related quality of life in different clinical settings, as well as for general populations. The SF-36 is divided into 8 multi-item scales, namely Physical Functioning (10 items), Role Limitations due to Physical Health (4 items), Role Limitations due to Emotional Problems (3 items), Energy/Fatigue (4 items), Emotional Well-Being (5 items), Social Functioning (2 items), Pain (2 items), and General Health Perception (5 items). The questions are scored on a Likert scale. Each of the summed scores is linearly transformed onto a scale from 0 (worse health) to 100 (better health) to provide a score for each subscale. Each subscale can be used independently. One item (the 36th) can be used to separately evaluate changes in health status compared to the previous year.

The assessment of both the patient’s dietary habits and adherence to the Mediterranean diet was conducted using the questionnaire validated by the “PREvención con DIeta MEDiterránea” (PREDIMED) trial [17]. The tool comprises 14 questions regarding food groups to include in the diet: olive oil, vegetables, fruits, meat and processed meats, dairy products, sugary and carbonated drinks, legumes, fish, alcohol, sweets, and nuts. Each question has two possible answers, with a score of 0 or 1 assigned to each. By summing the results, the level of adherence to the Mediterranean diet is determined as follows:

- Low adherence: score less than 5;
- Medium adherence: score between 6 and 9;
- High adherence: score equal to or greater than 10.

The last section included an open-ended question: “What is the main issue you feel you need help with?” This question aimed to allow the patient to express to the healthcare provider any difficulties encountered in their clinical and psychological journey related to their condition as cancer survivors.

### 2.4. Statistical Analysis

Information derived from the data of the questionnaires was automatically saved in a CSV file and then cleaned using the Statistical Package for Social Sciences (SPSS) version 26. The variables were described using frequencies and percentages, mean and median, and standard deviations (SDs). Information was reported for total and for age groups (under and over 65 years old). Comparisons in multiple co-morbidities between mean age groups were examined by Pearson’s chi-squared test. The effect size was determined by odd ratios (CI 95%). An alpha level of 0.05 was used for all statistical analyses. Non-parametric Mann–Whitney U Test for independent samples was used to investigate differences in questionnaires’ answers between age groups. The significance level was set at *p* < 0.05.

## 3. Results

A total of 62 former patients (80.6% females) agreed to be interviewed and signed the informed consent form; 16% of approached eligible participants declined their participation after the purpose of the study was explained.

Out of the total, 75.8% (n. 47) had former breast cancer, and 24.2% (n. 15) had colorectal cancer. In total, 54.5% (n. 34 persons) were treated with chemotherapy, 54.5% (n. 34 persons) with radiotherapy, and 62.9% (39 people) were treated with hormone therapy. Only seven people (11.1%) received only surgery.

The mean age was 64.9 years (years range: 37–87), with a slightly higher prevalence of adults younger than 65 years (51.6%). The mean BMI was 25.9 ± 4.7 SD, and more than half of the people in the sample (56.5%) were malnourished, classified either as underweight (4.8%), overweight (33.9%), or obese (17.8%). As for living conditions, 12.9% of the clients interviewed reported being widowed, and 17.7% living alone.

Most participants had a stable emotional relationship, noted as cohabiting with their partner (69.3%) or with family members (11.3%). However, 17.7% of the interviewed reported living alone. They were mainly secondary- and high-school graduates (more than 70%), with also more than 20% having a university degree. Nearly 40% of the people in the sample were primary-school graduates or undergraduates.

Additional participant socio-demographic characteristics are presented in Table 1.

The most commonly self-reported co-morbidities among participants were arthrosis, reported by more than half of the sample (61.3%), followed by hypertension, reported by nearly half of the sample (45.2%). Dental problems were reported by one-third of the clients (32.3%). Gastrointestinal diseases (25.8%), vascular diseases (25.8%), urological diseases (21.0%), diabetes (19.4%), and endocrinal diseases (19.4%) were declared by more than a quarter of the people interviewed. Other health conditions less prevalent in the sample are shown in Table 2. When splitting by age group, older people (>65 years) were more affected by all types of co-morbidities, with the only exception for subsequent cancer, anemia, and skin diseases reported mainly by the youngest group. The same analysis was made according to gender; females were more affected by all types of co-morbidities, with the only exception for kidney diseases, glaucoma, and stroke (Appendix A, Table A1).

As for the total, the mean number of co-morbidities self-reported was 3.6 ± 2.4 SD, with a statistically significant difference between the two age groups: 2.65 ± 1.71 SD for ≤65 years old vs. 4.73 ± 2.62 SD for people aged >65 years (*p* < 0.001). The risk of having multimorbidity (four or more co-morbidities) significantly increased in those over 65 years old (OR: 4.72; CI: 1.43–15.59; *p* = 0.008)

Table 3 shows the type of services (either health or social services) that participants were benefiting from. The most common answers were those for social services, specifically volunteers from non-governmental organizations (82.3%), followed by old people’s centers (79.0%).

Table 4 shows the scores for the different questionnaire areas (physical condition, mental condition, functional capabilities, support from the community, and socio-economic condition), as well as for the total. Each score has been presented also by age sub-group. Compared to their counterparts, older clients presented a statistically significantly worse functional capacity. Differences between two age groups were observed for all the other items, as well as for the total score, despite the results not being statistically significant. The same investigation was made by gender, but no statistically significant results were reported.

According to the evaluation scale, 8.1% of the sample was frail, 17.7% was pre-frail, and 74.2% was robust.

Table 5 shows the values of the SF-36 dimensions’ scores for total and by age group. Considering the overall sample, among all scales, PF, SF, and RE had the highest scores (>70%). Contrariwise, the lower scores were registered for VT and GH (≤60%). Generally, the quality of life was reduced when age increased, with the youngest group having better scores than the oldest group for all the items. The statistically significant differences between the two age groups were reported for PF (73.93 ± 28.61 vs. 84.06 ± 24.22; *p* < 0.001) and RP (77.34 ± 33.21 vs. 54.17 ± 47.38; *p* = 0.04) and for the total questionnaire score (73.18 ± 18.08 vs. 61.20 ± 20.78; *p* = 0.03).

The same investigation was made according to gender, but no statistically significant results were reported.

**Figure 1:**
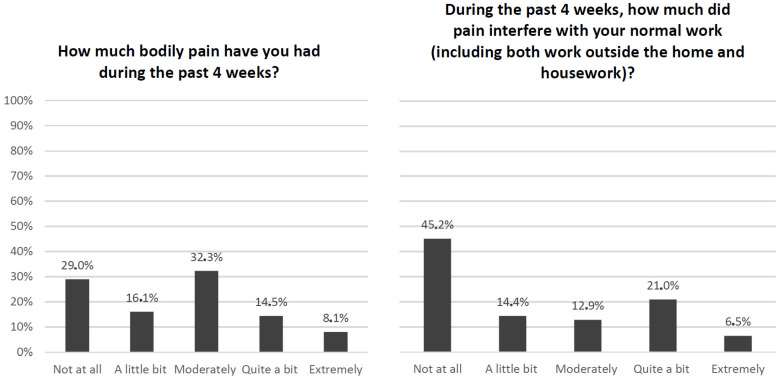
SF-36 questions on bodily pain during the past 4 weeks (questions no. 20 and no. 21).

**Figure 2:**
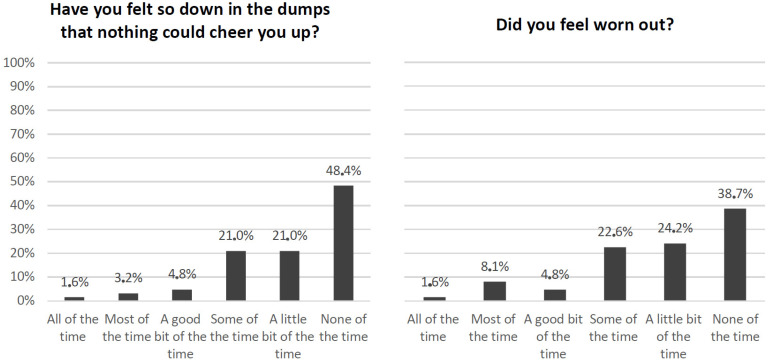
SF-36 questions about feeling and how things have been during the past 4 weeks (questions no. 25 and no. 29).

From the eight scales of the SF-36 questionnaire, we specifically identified key questions to depict the physical component and the mental component of cancer survivors (Figure 1 and Figure 2).

In Figure 1 are reported the two conditions concerning bodily pain that could have deeply impacted patients’ quality of life. When asked the first question, “How much bodily pain have you had during the last 4 weeks?” less than half of the respondents revealed either the absence of pain or scarce pain (29.0% and 16.1%, respectively), while more than half of the sample answered in the ranges of moderate (32.3%), quite a bit (14.5%), and extremely (8.1%). When investigating if the bodily pain during the previous 4 weeks had interfered with normal daily work and activities, slightly more than half of the sample reported those activities having been disrupted not at all (45.2%) or a little bit (14.4%), while the rest of clients declared bodily pain having interfered moderately (12.9%), quite a bit (21.0%), or extremely (6.5%).

When asked the question, “Have you felt so down in the dumps that nothing could cheer you up?” (Figure 2), only less than half of the sample answered “none of the time” (48.4%), while the rest experienced it a little bit of the time (21.0%) and some of the time (21.0%) at the same level, followed by “a good bit of the time” (4.8%), “most of the time (3.2%), and “all of the time” (1.6%), respectively. As regards feeling worn out, overall, more than one-third of respondents declared feeling it in a range from “some of the time” (22.6%) to “all of the time” (1.6%); only 24 people (38.7%) answered “none of the time”. These two conditions concerning mental health could have deeply impacted patients’ quality of life.

As regards the unmet needs, most of the sample (27.4%) complained about the management of the treatment path (which included smooth access to follow-up appointments and/or specific medical visits and diagnostics), and about practical help in daily life related to their specific health conditions (e.g., support in housekeeping). Ten clients declared no specific needs. Additional self-reported needs are listed in Table 6.

Table 7 shows that the mean value of the PREDIMED score was, overall, 8.58 ± 2.3 SD, with most of the sample following a proper Mediterranean regime, ranging from medium (59.7%) to good (32.3%) Mediterranean diet adherence. Only less than 10% of the sample had a poor PREDIMED score (8.1%).

**Figure 3:**
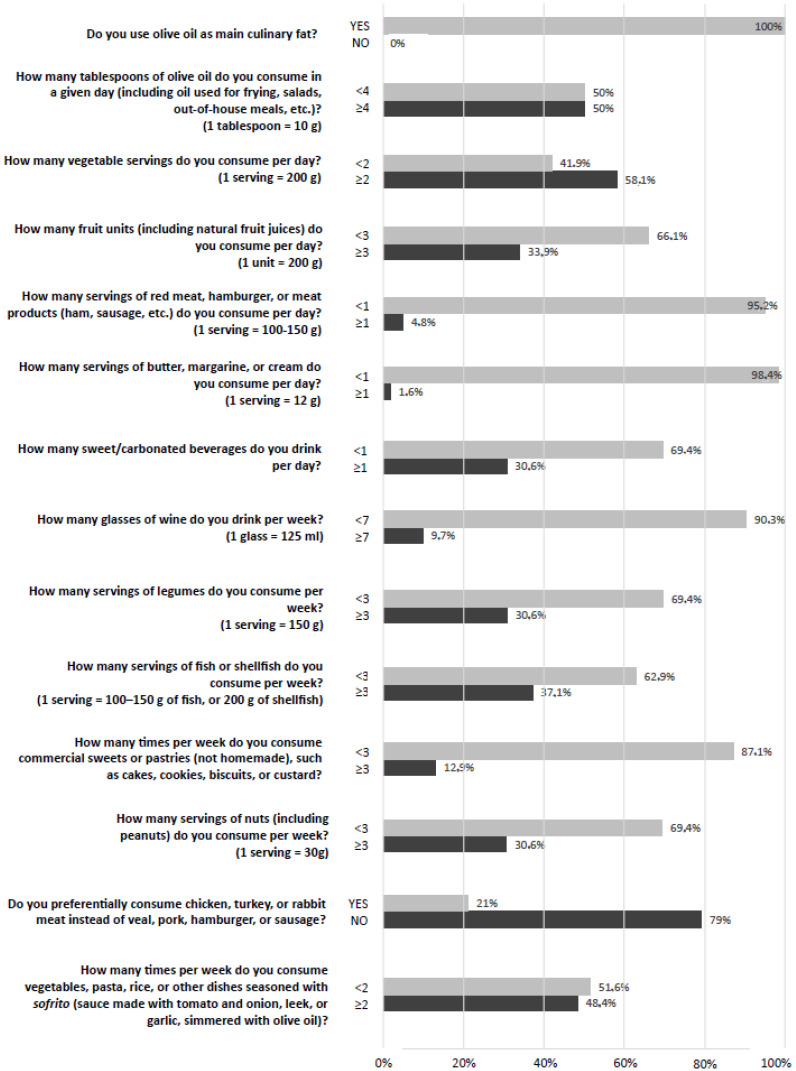
PREDIMED answers.

The specific answers to the 14 questions regarding food groups to include in the diet are presented in Figure 3.

## 4. Discussion

Although the survival of cancer patients has impressively improved in the last decades and is expected to increase further in the following years, little is still known about cancer survivors’ quality of life, frailty, co-morbidity incidence, probability of new cancers, or need for social and health services. To better understand the outcomes of the cancer survivor population, it is essential to assess different aspects and conditions of life that might be important for identifying specific long-term sequelae of cancer and treatments [18]. Some research has already been conducted in this field, underlining that problems faced by cancer survivors were related to medical care, psychological support, social support, and daily practical help [19,20].

Specifically, lower physical, mental, and emotional functioning, as well as higher fatigue levels, have been detected in many cancer survivors [21]. Following Kurtin and Fuoto [22], body pain is a primary challenge for cancer survivors across all age groups, and, in many cases, its management remains an unmet need. Our results are in line with these previous findings. Effective pain management requires an interdisciplinary approach tailored to each patient, essential to providing the continuum of care. In conjunction, cancer survivors should be screened for mental status, since psychological and emotional disorders suffered by many long-term survivors can be due to pain and fatigue [23,24]. From our findings, the high rate of people reporting bodily pain interfering with daily activities suggests that it could be related to the condition of cancer itself and its treatment. Following the present pilot, further studies are needed to conduct a more in-depth analysis to untangle the additional burden which can be attributed to cancer/cancer treatment.

Notably, Nardin et al. discovered that breast cancer survivors faced also substantial psychosocial hurdles, such as depression, anxiety, and intrusive thoughts; the authors underscored the pivotal role of social and family support in combating feelings of loneliness and isolation [25]. Similarly, Roine and his team revealed that younger survivors were more susceptible to depressive symptoms and emotional distress than their older counterparts [26]. However, our study yielded different results, showing that older individuals were more emotionally vulnerable than their younger counterparts. It is necessary to emphasize the importance of psychological support, as anxiety and depression are strongly associated with a lower quality of life [27], regardless of age.

Thong and colleagues highlighted that challenges faced by colorectal cancer survivors (such as difficulties in performing daily activities, altered emotional state, and pain) negatively affected the quality of life of cancer survivors and persisted even many years after diagnosis; therefore, the authors highlighted the need for comprehensive care planning [28] and a multidisciplinary approach to addressing long-term needs, as Fan and colleagues have also described [29].

Despite the above-mentioned outcomes, the need for multidimensional assessment and support of cancer survivors has not been enough emphasized, nor has the importance of holistic care that addresses medical, psychological, social, and practical aspects to improve the quality of life and well-being of cancer survivors. As a consequence, research currently does not provide sufficient information to enable evidence-based guidelines for cancer survivorship planning to be formed. Even in countries with more advanced approaches to survivors’ care, there are few guidelines on the management of cancer survivors’ needs.

With growing populations of cancer survivors, Italian clinical care, public policy, and research initiatives should prepare to respond to the health transition of patients diagnosed with cancer. This can be achieved by integrating data collection on the health, social, and mental conditions of cancer patients into a comprehensive treatment management.

A previous study conducted by Annunziata et al. [30] demonstrated that cancer survivors differed from both cancer patients and the general population. More specifically, they found that cancer survivors had higher scores of physical and mental functioning than those of cancer patients, but lower than those of the general population. Additionally, another of the few studies in Italy using questionnaire SF-36 showed more significant limitations in the daily life of adult cancer survivors, mainly due to worse physical functioning, physical and emotional problems, and less vitality than in the general adult population [31].

To the best of our knowledge, the present pilot study is one of the first multidimensional investigations with a multidisciplinary approach conducted in Italy. An original aspect of our study is the comprehensive assessment oriented to the frailty evaluation. Notably, this is the first pilot using a specific tool (the Grauer–Palombi questionnaire) to provide a comprehensive picture of the prevalence of frailty and factors associated with this condition among cancer survivors. 

Additionally, this is one of the first studies in which adherence to the Mediterranean diet, specifically using the PREDIMED questionnaire, was assessed in the adult cancer survivor population. In the current literature, only another study used this tool in women who survived breast cancer. However, as the assessment was conducted in a New Zealand population, the results might not be comparable [32]. The impact of dietary interventions on various aspects of the well-being of colorectal cancer survivors has been previously considered [33,34], and it is already well documented that obese patients have a significantly elevated risk for subsequent tumors [35,36]. In our study population, high rates of overweight and obesity have emerged, an interesting finding that deserves further detailed studies.

This investigation allows us to draft a picture of the main characteristics of an Italian cancer survivor: an overaged person with multiple co-morbidities, mainly presenting body weight excess despite adhering to the Mediterranean diet, and who is benefiting from social and health services. His/her main conditions are related to physical and functional status (i.e., body pain and feeling low). The older sub-sample displayed worse conditions for all the investigated items than younger participants. These findings can be interpreted in light of the role played by the primary aging process [30]. Since the incidence of chronic diseases and frailty conditions increases with age, so care models must be adapted to a triple burden that combines cancer survivorship, chronic diseases [37], and multimorbidity [38].

Additionally, cancer survivors may develop more health problems due to the adverse effects of cancer treatment or complications of cancer itself [39]. The most frequently reported co-morbidities in our population were in line with findings from a systematic review [40]. Several studies have investigated adverse health consequences from cancer itself and its treatment, finding multimorbidity to be up to three times higher in people living with and beyond cancer compared to the general population [41]. In this pilot study, the prevalence of the main conditions was compared with the most recent available data for the adult general population [42], as well as for those over 65 years old [43] at the national level. Individuals in our population showed higher rates of arthrosis (61.3% in the current study vs. 14.8% in the general adult population), especially when over 65 years old (76.7% in the current study vs. 47.6% in the general elderly population); hypertension for the overall sample (45.2% vs. 18.8%), most especially in elderly (66.7% vs. 47%); diabetes (19.4% vs. 5.9%), most especially in elderly (30.0% vs. 16.8%); asthma (11.3% vs. 5.8%); and cardiac disease (16.1% vs. 4.2%), most especially in over 65 (26.7% vs. 19.3%). In previous studies, cardiovascular risk factors (such as hypertension, diabetes, and obesity) have already been registered significantly higher in cancer survivors than in comparative populations [44]. Generally, 80.7% of the sample reported two or more co-morbidities (multimorbidity); this rate was in line with the prevalence of multimorbidity registered in other studies conducted on adult cancer survivors (multimorbidity range: 23.6–82.7%) [40]. As regards the incidence of subsequent/secondary cancer registered, especially in the younger group, most of the information included secondary benign tumors (for example, meningioma) and/or non-malignant syndrome.

Lastly, our findings underline that the two major self-reported unmet needs were the management of the treatment pathway during the transition from oncological to primary care and the practical help requested in daily life activities related to specific health conditions. We acknowledge that improvement in the healthcare pathway and social support for cancer survivors is still needed [45]. However, in some other studies, access to care and preventive services has generally been reported as equivalent or more significant compared to other individuals [46].

### Limitations

The small sample size of this pilot is one of the main limitations of the study. Additionally, we acknowledge the absence of data on key cancer characteristics, including stage, type of anti-cancer treatment, and its duration, that could have influenced the results; consequently, the potential effects of those factors on life quality could not be accounted for.

Secondly, there were more female participants than males in the enrolled sample, and breast cancer was the predominant diagnosis; thus, these facts may have influenced the patterns observed, and we should take that into account in interpreting these data.

All information was self-reported, including health conditions and co-morbidities, so the measure of multimorbidity could have overestimated the conditions experienced by the study participants. This should be taken especially into account when considering the high incidence of subsequent/secondary tumors. However, in most of the studies analyzed, multimorbidity was self-reported [40].

Additionally, we acknowledge the absence of a control-group study; a comparison to controls could have allowed us to identify conditions where cancer survivors may benefit from additional surveillance. Therefore, when available, our data were compared with those of the general population.

Lastly, further studies are needed to conduct a more in-depth analysis to untangle the additional burden which can be attributed to cancer/cancer treatment, especially when considering the high rate of people reporting bodily pain.

## 5. Conclusions

The healthcare sector for cancer survivors necessitates adopting a person-centered participatory approach for a continuum of care that incorporates investigation, assessment, and monitoring of specific needs (i.e., physical, psychological, emotional, social, and health), undergoing follow-up care, and cancer surveillance.

Data provided by the current study show how the quality of life of cancer survivors has been affected by cancer and its treatment. Multimorbidity was highly prevalent and one of the main concerns among cancer survivors, together with emotional and physical challenges. Therefore, identifying risk factors, specific needs, and worrying conditions may be beneficial to improve the general quality of life among cancer survivors.

Our findings may be relevant to developing survivorship care planning for the transition of individuals from the oncological setting back to management by primary-care physicians. A multidisciplinary coordinated care model would facilitate appropriate, tailored person-based responses. Further studies should assess the designed interventions.

## Figures and Tables

**Table 1:** Socio-demographic characteristics.

Variables	n (%)
**Gender**	
Male	12 (19.4)
Female	50 (80.6)
**Mean age**	64.9 years ± 10.8 SD
**Median age**	65.0 years
**Age range (min–max)**	37–87 years
**Age group**	
≤65	32 (51.6)
>65	30 (48.4)
**Mean BMI**	25.9 ± 4.7 SD
**Median BMI**	25.0
**BMI range (min–max)**	18–40
BMI group classes	
Underweight (<18.5)	3 (4.8)
Normal weight (18.5–24.9)	27 (43.5)
Overweight (25–29.9)	21 (33.9)
Obesity (≥30)	11 (17.8)
**Marital status**	
**Married**	43 (69.4)
Single/divorced	11 (17.8)
Widowed	8 (12.9)
**Living conditions**	
Alone	11 (17.7)
With partner	43 (69.3)
With family members	7 (11.3)
With caregiver	1 (1.6)
**Education**	
Primary school	4 (6.5)
Secondary school	22 (35.5)
High school	22 (35.5)
University	14 (22.6)
**Area of residence**	
Urban	57 (91.9)
Rural	5 (8.1)

**Table 2:** Self-reported health conditions, for total and by age groups.

Type of Co-Morbidity	n (%)
**Arthrosis**	38 (61.3)
≤65 years	15 (46.9)
>65 years	23 (76.7)
**Hypertension**	28 (45.2)
≤65 years	8 (25.0)
>65 years	20 (66.7)
**Dental problem**	20 (32.3)
≤65 years	9 (28.1)
>65 years	11 (36.7)
**Subsequent/secondary cancer**	18 (29.0)
≤65 years	11 (34.4)
>65 years	7 (23.3)
**Gastro-intestinal diseases**	16 (25.8)
≤65 years	7 (21.9)
>65 years	9 (30.0)
**Vascular diseases**	16 (25.8)
≤65 years	4 (12.5)
>65 years	12 (40.0)
**Urologic diseases**	13 (21.0)
≤65 years	2 (6.3)
>65 years	11 (36.7)
**Diabetes**	12 (19.4)
≤65 years	3 (9.4)
>65 years	9 (30.0)
**Endocrine diseases**	12 (19.4)
≤65 years	6 (18.8)
>65 years	6 (20.0)
**Cardiac diseases**	10 (16.1)
≤65 years	2 (6.3)
>65 years	8 (26.7)
**Anemia**	9 (14.5)
≤65 years	5 (15.6)
>65 years	4 (13.3)
**Emphysema**	8 (12.9)
≤65 years	3 (9.4)
>65 years	5 (16.7)
**Asthma**	7 (11.3)
≤65 years	4 (12.5)
>65 years	3 (10.0)
**Liver diseases**	6 (9.7)
≤65 years	2 (6.3)
>65 years	4 (13.3)
**Neurologic diseases**	4 (6.5)
≤65 years	2 (6.3)
>65 years	2 (6.7)
**Kidney diseases**	3 (4.8)
≤65 years	0
>65 years	3 (10.0)
**Skin diseases**	3 (4.8)
≤65 years	2 (6.3)
>65 years	1 (3.3)
**Peptic ulcer**	2 (3.2)
≤65 years	0
>65 years	2 (6.7)
**Glaucoma**	1 (1.6)
≤65 years	0
>65 years	1 (3.3)
**Stroke**	1 (1.6)
≤65 years	0
>65 years	1 (3.3)
**Multimorbidity (no. of conditions)**	
None	2 (3.2)
One	10 (16.1)
Two	9 (14.5)
Three	12 (19.4)
Four	10 (16.1)
Five	7 (11.3)
Six	8 (12.9)
Nine	3 (4.8)
Thirteen	1 (1.6)

**Table 3:** Beneficiaries from health and social services.

Type of Services	n (%)
A nurse from the health service	44 (71.0)
Homecare helps from the health service	48 (77.4)
Volunteers from non-governmental organizations	51 (82.3)
Old people’s centers	49 (79.0)

**Table 4:** Grauer–Palombi scale results.

Item	Mean ± SD	Signif.
**Physical condition**	−3.32 ± 7.49	
≤65 years old	−1.75 ± 4.27	0.11
>65 years old	−5.00 ± 9.63	
**Mental condition**	−0.81 ± 1.98	
≤65 years old	−0.72 ± 1.72	0.89
>65 years old	−0.90 ± 2.25	
**Functional capacity**	39.21 ± 5.46	
≤65 years old	40.41 ± 2.41	**0.03**
>65 years old	37.93 ± 7.30	
**Support from the community**	27.98 ± 8.06	
≤65 years old	28.78 ± 7.31	0.62
>65 years old	27.13 ± 8.83	
**Household and economic condition**	13.50 ± 5.13	
≤65 years old	14.06 ± 4.79	0.70
>65 years old	12.90 ± 5.49	
**Grauer–Palombi total score**	76.98 ± 15.86	
≤65 years old	80.41 ± 11.25	0.40
>65 years old	73.33 ± 19.17	
**Grauer–Palombi classification**	**n (%)**	
Frail	5 (8.1)	
Pre-frail	11 (17.7)	
Robust	46 (74.2)	

**Table 5:** SF-36 questionnaire’s results.

Item	Mean ± SD	Signif.
**Physical function (PF)**	73.93 ± 28.61	
≤65 years old	84.06 ± 24.22	**<0.001**
>65 years old	62.75 ± 29.08	
**Limitations due to physical problem (RP)**	66.12 ± 42.00	
≤65 years old	77.34 ± 33.21	**0.046**
>65 years old	54.17 ± 47.38	
**Limitations due to emotional problem (RE)**	72.04 ± 39.67	
≤65 years old	82.29 ± 29.31	0.07
>65 years old	61.11 ± 23.26	
**Vitality, energy, and fatigue (VT)**	60.82 ± 20.88	
≤65 years old	61.56 ± 23.26	0.778
>65 years old	60.00 ± 18.27	
**General mental health (MH)**	66.38 ± 17.07	
≤65 years old	69.50 ± 17.08	0.09
>65 years old	63.07 ± 16.69	
**Social activities (SF)**	73.99 ± 21.74	
≤65 years old	78.90 ± 20.44	0.07
>65 years old	68.75 ± 22.20	
**Bodily pain (BP)**	68.22 ± 28.03	
≤65 years old	71.40 ± 28.83	0.37
>65 years old	64.83 ± 27.22	
**General health (GH) perceptions**	59.74 ± 20.00	
≤65 years old	62.09 ± 22.75	0.32
>65 years old	57.14 ± 16.46	
**SF-36 total score**	67.71 ± 20.10	
≤65 years old	73.18 ± 18.08	**0.03**
>65 years old	61.20 ± 20.78	

**Table 6:** Specific self-reported unmet needs.

Needs	n (%)
Management of the treatment path	17 (27.4)
Practical daily help related to specific health conditions	16 (25.8)
None	10 (16.1)
Specialized medical assistance	6 (9.7)
Psychological support	2 (3.2)
Social support	1 (1.6)
Other needs (economical)	1 (1.6)

**Table 7:** PREDIMED results.

Mediterranean Diet Adherence	n (%)
Poor (≤5)	5 (8.1)
Medium (6–9)	37 (59.7)
Good (≥10)	20 (32.3)
**PREDIMED (Mean ± SD)**	8.58 ± 2.3

## Data Availability

Data will be made available upon reasonable request.

## Appendix A

**Table A1:** Self-reported health conditions by gender.

Co-Morbidity	n (%)
**Arthrosis**	**38 (61.3)**
Males	4 (10.5)
Females	34 (89.5)
**Hypertension**	**28 (45.2)**
Males	7 (25.0)
Females	21 (75.0)
**Dental problem**	**20 (32.3)**
Males	3 (15.0)
Females	17 (85.0)
**Subsequent/secondary cancer**	**18 (29.0)**
Males	2 (11.1)
Females	16 (88.9)
**Gastro-intestinal diseases**	**16 (25.8)**
Males	2 (12.5)
Females	14 (87.5)
**Vascular diseases**	**16 (25.8)**
Males	2 (12.5)
Females	14 (87.5)
**Urologic diseases**	**13 (21.0)**
Males	4 (30.8)
Females	9 (69.2)
**Diabetes**	**12 (19.4)**
Males	3 (25.0)
Females	9 (75.0)
**Endocrine diseases**	**12 (19.4)**
Males	0 (0)
Females	12 (100)
**Cardiac diseases**	**10 (16.1)**
Males	2 (20.0)
Females	8 (80.0)
**Anemia**	**9 (14.5)**
Males	2 (22.2)
Females	7 (77.8)
**Emphysema**	**8 (12.9)**
Males	3 (37.5)
Females	5 (62.5)
**Asthma**	**7 (11.3)**
Males	1 (14.3)
Females	6 (85.7)
**Liver diseases**	**6 (9.7)**
Males	3 (50.0)
Females	3 (50.0)
**Neurologic diseases**	**4 (6.5)**
Males	2 (50.0)
Females	2 (50.0)
**Kidney diseases**	**3 (4.8)**
Males	2 (66.7)
Females	1 (33.3)
**Skin diseases**	**3 (4.8)**
Males	0 (0)
Females	3 (100)
**Peptic ulcer**	**2 (3.2)**
Males	0 (0)
Females	2 (100)
**Glaucoma**	**1 (1.6)**
Males	1 (100)
Females	0 (0)
**Stroke**	**1 (1.6)**
Males	1 (100)
Females	0 (0)

## References

[1] Baili P., Vicentini M., Tumino R., Vercelli M., Lorenzo M., Foschi R., Guzzinati S., Dal Maso L., Minicozzi P., de Lorenzo F. (2013). A method for differentiating cancer prevalence according to health status, exemplified using a population-based sample of Italian colorectal cancer cases. Acta Oncol..

[2] De Angelis R., Grande E., Inghelmann R., Francisci S., Micheli A., Baili P., Meneghini E., Capocaccia R., Verdecchia A. (2007). Cancer prevalence estimates in Italy from 1970 to 2010. Tumori.

[3] Rapporto “I numeri del cancro in Italia 2023”, Associazione Italiana di Oncologia Medica (AIOM), Associazione Italiana Registri Tumori (AIRTUM), Fondazione AIOM, Osservatorio Nazionale Screening (ONS), sorveglianze di popolazione PASSI (Progressi delle Aziende Sanitarie per la Salute in Italia) e PASSI d’Argento e Società Italiana di Anatomia Patologica e di Citologia Diagnostica (SIAPEC-IAP). https://www.aiom.it/wp-content/uploads/2023/12/2023_AIOM_NDC-web.pdf.

[4] Marzorati C., Riva S., Pravettoni G. (2017). Who Is a Cancer Survivor? A Systematic Review of Published Definitions. J. Cancer Educ..

[5] Smith A.W., Reeve B.B., Bellizzi K.M., Harlan L.C., Klabunde C.N., Amsellem M., Bierman A.S., Hays R.D. (2008). Cancer, comorbidities, and health-related quality of life of older adults. Health Care Financ. Rev..

[6] Bamonti P.M., Moye J., Naik A.D. (2018). Pain is associated with continuing depression in cancer survivors. Psychol Health Med..

[7] Liu W.C., Zheng Z.X., Tan K.H., Meredith G.J. (2017). Multidimensional treatment of cancer pain. Curr. Oncol. Rep..

[8] Hewitt M., Greenfield S., Stovall E. (2006). From Cancer Patient to Cancer Survivor: Lost in Transition.

[9] Groen W.G., Kuijpers W., Oldenburg H.S., Wouters M.W., Aaronson N.K., van Harten W.H. (2015). Empowerment of cancer survivors through information technology: An integrative review. J. Med. Internet Res..

[10] Lustberg M.B., Kuderer N.M., Desai A., Bergerot C., Lyman G.H. (2023). Mitigating long-term and delayed adverse events associated with cancer treatment: Implications for survivorship. Nat. Rev. Clin. Oncol..

[11] Salz T., Oeffinger K.C., McCabe M.S., Layne T.M., Bach P.B. (2012). Survivorship care plans in research and practice. CA Cancer J. Clin..

[12] Gallicchio L., Tonorezos E., de Moor J.S., Elena J., Farrell M., Green P., Mitchell S.A., Mollica M.A., Perna F., Saiontz N.G. (2021). Evidence gaps in cancer survivorship care: A report from the 2019 National Cancer Institute Cancer Survivorship Workshop. J. Natl. Cancer Inst..

[13] Barbui T., Björkholm M., Gratwohl A. (2014). Cancer survivorship programs: Time for concerted action. Haematologica.

[14] Scarcella P., Liotta G., Marazzi M.C., Carbini R., Palombi L. (2005). Analysis of survival in a sample of elderly patients from Ragusa, Italy on the basis of a primary care level multidimensional evaluation. Arch. Gerontol. Geriatr..

[15] Grauer H., Birnbom F. (1975). A geriatric functional rating scale to determine the need for institutional care. J. Am. Geriatr. Soc..

[16] Ware J.E., Sherbourne C.D. (1992). The MOS 36-item short-form health survey (SF-36). I. Conceptual framework and item selection. Med. Care.

[17] Martínez-González M.A., García-Arellano A., Toledo E., Salas-Salvadó J., Buil-Cosiales P., Corella D., Covas M.I., Schröder H., Arós F., Gómez-Gracia E. (2012). A 14-item Mediterranean diet assessment tool and obesity indexes among high-risk subjects: The PREDIMED trial. PLoS ONE.

[18] Mosconi P., Apolone G., Barni S., Secondino S., Sbanotto A., Filiberti A. (2002). Quality of life in breast and colon cancer long-term survivors: An assessment with the EORTC QLQ-C30 and SF-36 questionnaires. Tumori.

[19] Doege D., Thong M.S.Y., Weißer L., Koch-Gallenkamp L., Jansen L., Bertram H., Eberle A., Holleczek B., Nennecke A., Pritzkuleit R. (2021). Health-Related Quality of Life in Very Long-Term Cancer Survivors 14–24 Years Post-Diagnosis Compared to Population Controls: A Population-Based Study. Cancers.

[20] Schmidt M.E., Goldschmidt S., Hermann S., Steindorf K. (2022). Late effects, long-term problems and unmet needs of cancer survivors. Cancer Epidemiol..

[21] Faithfull S., Greenfield D. (2024). Cancer survivor late-effects, chronic health problems after cancer treatment: What’s the evidence from population and registry data and where are the gaps?. Curr. Opin. Support. Palliat. Care.

[22] Kurtin S., Fuoto A. (2019). Pain Management in the Cancer Survivor. Semin. Oncol. Nurs..

[23] Al-Shandudi M., Al-Mandhari M., Chan M.F., Al-Hajri T., Al-Balushi M., Al-Azri M. (2022). Health-Related Quality of Life of Omani Colorectal Cancer Survivors. Cancer Control.

[24] Liang Y., Zhang X., Li S., Wang Z. (2024). Comparison of psychological interventions for anxiety, depression, fatigue and quality of life in colorectal cancer survivors: A systematic review and network meta-analysis protocol. PLoS ONE.

[25] Nardin S., Mora E., Varughese F.M., D’Avanzo F., Vachanaram A.R., Rossi V., Saggia C., Rubinelli S., Gennari A. (2020). Breast Cancer Survivorship, Quality of Life, and Late Toxicities. Front Oncol..

[26] Roine E., Sintonen H., Kellokumpu-Lehtinen P.L., Penttinen H., Utriainen M., Vehmanen L., Huovinen R., Kautiainen H., Nikander R., Blomqvist C. (2021). Long-term health-related quality of life of breast cancer survivors remains impaired compared to the age-matched general population especially in young women. Results from the prospective controlled BREX exercise study. Breast.

[27] Park J., Rodriguez J.L., O’Brien K.M., Nichols H.B., Hodgson M.E., Weinberg C.R., Sandler D.P. (2021). Health-related quality of life outcomes among breast cancer survivors. Cancer.

[28] Thong M.S.Y., Doege D., Weißer L., Koch-Gallenkamp L., Jansen L., Bertram H., Eberle A., Holleczek B., Nennecke A., Waldmann A. (2023). Persisting deficits in health-related quality of life of colorectal cancer survivors 14–24 years post-diagnosis: A population-based study. Curr. Oncol..

[29] Fan R., Wang L., Bu X., Wang W., Zhu J. (2023). Unmet supportive care needs of breast cancer survivors: A systematic scoping review. BMC Cancer.

[30] Annunziata M.A., Muzzatti B., Giovannini L., Romito F., Cormio C., Mattioli V., Barberio D., Abate V., De Falco F., Mirabella F. (2015). Is long-term cancer survivors’ quality of life comparable to that of the general population? An Italian study. Support Care Cancer.

[31] Annunziata M.A., Muzzatti B., Flaiban C., Gipponi K., Carnaghi C., Tralongo P., Caruso M., Cavina R., Tirelli U. (2018). Long-term quality of life profile in oncology: A comparison between cancer survivors and the general population. Support Care Cancer.

[32] Braakhuis A., Campion P., Bishop K. (2017). The Effects of Dietary Nutrition Education on Weight and Health Biomarkers in Breast Cancer Survivors. Med. Sci..

[33] Jung Y., Chung J., Son H. (2020). Physical Activity Interventions for Colorectal Cancer Survivors: A Systematic Review and Meta-analysis of Randomized Controlled Trials. Cancer Nurs..

[34] Ho M., Ho J.W.C., Fong D.Y.T., Lee C.F., Macfarlane D.J., Cerin E., Lee A.M., Leung S., Chan W.Y.Y., Leung I.P.F. (2020). Effects of dietary and physical activity interventions on generic and cancer-specific health-related quality of life, anxiety, and depression in colorectal cancer survivors: A randomized controlled trial. J. Cancer Surviv..

[35] Park S.M., Lim M.K., Jung K.W., Shin S.A., Yoo K.Y., Yun Y.H., Huh B.Y. (2007). Prediagnosis smoking, obesity, insulin resistance, and second primary cancer risk in male cancer survivors: National Health Insurance Corporation Study. J. Clin. Oncol..

[36] Jung S.Y., Kim Y.A., Jo M., Park S.M., Won Y.J., Ghang H., Kong S.Y., Jung K.W., Lee E.S. (2019). Prediagnosis obesity and secondary primary cancer risk in female cancer survivors: A national cohort study. Cancer Med..

[37] Leach C.R., Weaver K.E., Aziz N.M., Alfano C.M., Bellizzi K.M., Kent E.E., Forsythe L.P., Rowland J.H. (2015). The complex health profile of long-term cancer survivors: Prevalence and predictors of comorbid conditions. J. Cancer Surviv..

[38] Nicholson K., Terry A.L., Fortin M., Williamson T., Thind A. (2015). Understanding multimorbidity in primary health care. Can. Fam. Physician.

[39] Andresen K., Carreira H., Strongman H., McDonald H.I., Benitez-Majano S., Mansfield K.E., Nitsch D., Tomlinson L.A., Bhaskaran K. (2023). The risk of acute kidney injury in colorectal cancer survivors: An english population-based matched cohort study. BMC Cancer.

[40] Asogwa O.A., Quansah D.Y., Boakye D., Ezewuiro O.N., Boateng D. (2023). Prevalence, patterns, and determinants of multimorbidity among childhood and adult cancer survivors: A systematic review. Crit. Rev. Oncol. Hematol..

[41] Ahmad T.A., Gopal D.P., Chelala C., Dayem Ullah A.Z., Taylor S.J. (2023). Multimorbidity in people living with and beyond cancer: A scoping review. Am. J. Cancer Res..

[42] ISTAT (2022). Annuario Statistico Italiano 2022.

[43] Report ISTAT Le Condizioni di Salute della Popolazione Anziana in Italia—Anno 2019. https://www.istat.it/wp-content/uploads/2021/07/Report-anziani-2019.pdf.

[44] Koric A., Chang C.P., Mark B., Rowe K., Snyder J., Dodson M., Deshmukh V.G., Newman M.G., Fraser A.M., Smith K.R. (2022). Cardiovascular disease risk in long-term breast cancer survivors: A population-based cohort study. Cancer.

[45] Nicoll I., Lockwood G., Fitch M.I. (2023). Perspectives of Cancer Survivors with Low Income: A Content Analysis Exploring Concerns, Positive Experiences, and Suggestions for Improvement in Survivorship Care. Curr. Oncol..

[46] Robin Yabroff K., Short P.F., Machlin S., Dowling E., Rozjabek H., Li C., McNeel T., Ekwueme D.U., Virgo K.S. (2013). Access to preventive health care for cancer survivors. Am. J. Prev. Med..

